# Notes of Epidemic Sore Throat (Diphtherite,) as Prevalent in Albany, N. Y.

**Published:** 1859-01

**Authors:** 


*Notes of Epidemic Sore Throat (Diphtherite,) as Prevalent in Albany, N. Y.* By S. D. Willard, M. D.

For four months past there has been a strong predisposition to affections of the throat in this community. These affections produced, doubtless, by the same epidemical influences, have existed under well defined and distinct varieties.

The first, and by far the most common form of the disease is *Pharyngitis.* It is a diffused inflammation covering the palate, uvula and tonsils, which become highly vascular, and give rise to a sensation of dryness and roughness in the fauces. The general health and appetite is undisturbed, and the only treatment required, is one or tw applications of nitrate of silver, or an astringent gargle. There have been hundreds of cases of this mild form, which in severity, has been scarcely sufficient to style disease.

The next variety is *sloughing tonsillitis.* It exists more particularly among children and young persons—those under twenty years of age. Upon looking into the fauces, it is at once observable, that the tonsils are swollen, in some cases so as nearly to touch each other, and on their surface, are white spots, in siz varying from a shot to a half-dime. This high degree of inflammation nnd suppurative process, comes on suddenly, and its progress is through in eight or ten days. These white ulcers have thick edges, and look deep seated. They become more extensive, involving the whole of the tonsils; but in most instances, the slough is thrown off, and resolution ensues. In a few cases, the tonsils have been of a dark mahogany color, and the ulcers assume a greenish cast, and have been followed by gangrene, mortification, and consequently death. In some of these cases of sloughing tonsillitis, there has been a psuedo-membrane upon the roof of the mouth, the palate, uvula and tonsils, which, by the process of suppuration, has been detached and thrown off.

The third and most fatal variety is *diphthérite.* This has prevailed mostly among children under seven years of age. Its onset is sudden and insidious. The false membrane usually having been formed when the first symptoms of illness attracted the attention, and occasionally, when the attention was directed only by the alarming condition of other children of the family. The membrane rapidly extends upon the palate, tonsils, the rima glottis, and into the larynx, producing mechanical obstruction to respiration, as in croup, and the patient dies in precisely the same manner.

There is yet a fourth, which if not a distinct variety, is at least a modification of all of them. It is styled by a medical friend of mine, in expressive language, "*the horse distemper variety.*" In this, there seems to be a blood poison, and the mucous membrane of the nose, fauces and bronchi, throw off a thick, offensive acrid secretion, and there follows, before death, incipient mortification and decomposition. The congestion extends to the cellulai tissue and skin about the throat and chest. As in many of the cases of sloughing tonsillitis, the parotid glands become affected and swollen. In this variety, there is no false membrane. It cannot therefore be *diphthérite;* yet it is a malady co-existent with it. From this form of the disease, nearly all die. Of the three last forms, within three months, about fifty have died. It is difficult to estimate, accurately, the number of cases that have occurred. Of the first and mild form of the disease, doubtless there have been a thousand cases; most of which under less apprehensive circumstances, would never have come under the eye of the physician. The similarity of sloughing tonsillitis, and the sore throat of scarlatina maligna, is worthy of notice. The almost entire absence of scarlatina, for the three past months, and its prevalence the three months preceeding, is a fact that should not escape observation. Aside from the local treatment in sever, cases, the strongly marked tendency to debility and prostration, calls early for invigorating and strengthening remedies. In several families, two to four children have died of one form or other of the disease. My attention has been called to the greater prevalence of the *diphthérite* form, in the southern part of the city.

The disease *Diphthérite* has been accurately described by that eminent French pathologist, Mr. Brettonneau, as it prevailed at Tours, and by him recognized as a distinct disease, and embraces that form of malady here spoken of under the *third* variety. A full, clear, and vigorous article on this subject, from the pen of R. J. Fourgeaud, M. D., is published in the *Pacific Medical and Surgical Jourual,* (San Francisco, California,) for October, 1858. The disease known as *depthérite,* or membranous sore throat, having prevailed in the valley of Sonoma, California, in 1856.

The epidemic in Albany is subsiding.—*Med. and Surg. Reporter.*

